# Prolonged Incubation Period for *Cryptococcus gattii* Infection in Cat, Alaska, USA

**DOI:** 10.3201/eid1906.130006

**Published:** 2013-06

**Authors:** Louisa J. Castrodale, Robert F. Gerlach, Diane E. Preziosi, Paul Frederickson, Shawn R. Lockhart

**Affiliations:** Alaska Division of Public Health, Anchorage, Alaska, USA (L.J. Castrodale);; Alaska Division of Environmental Health, Anchorage (R.F. Gerlach);; Veterinary Specialists of Alaska, PC, Anchorage (D.E. Preziosi);; Hillside Pet Clinic, Anchorage (P. Frederickson);; Centers for Disease Control and Prevention, Atlanta, Georgia, USA (S.R. Lockhart)

**Keywords:** Cryptococcus gattii, incubation period, infection, cat, fungi, California, Alaska

**To the Editor:** We report a case of *Cryptococcus gattii* infection in a 12-year-old neutered male cat in Alaska. The cat traveled to Anchorage, Alaska (61°N) from San Diego County, California, with its owner in August 2003. Although *C. gattii* has not been detected in Alaska (where only extremely limited sampling has occurred) or above 49°N (i.e., Vancouver Island, British Columbia), the case suggests that the incubation period for *C. gattii* could be >8 years.

In September 2010, the cat was brought to a veterinary clinic in Anchorage because of facial pruritis and excoriation. The cat did not respond to treatment with methylprednisolone acetate and was referred to a veterinary dermatologist in October. At that time, alopecia, thick scaling, and excoriations were observed on ear margins, sides of the head, and between the eyes. The hair coat was sparse, and there was minimal scaling near the tail and lower legs. All foot pads were excessively cross-hatched and scaly. Cytologic analysis of the skin on the head and pinna showed neutrophils and cocci overgrowth. Skin scrapings were negative for mites. Cytologic analysis of the ears did not identify yeast bodies, parasites, or bacteria. A long-acting antimicrobial drug for treatment of skin infections (cefovecin) was given.

Biopsy specimens were obtained from the head, ears, and paws, and analysis of these samples supported a diagnosis of mural folliculitis and mild plasma cell pododermatitis. Chest radiograph findings and results of routine blood analysis were not unusual. Test results for feline leukemia virus and feline immunodeficiency virus were negative. Prednisolone (1.8 mg/kg/d) was given; the cat showed a good response and eventual resolution of scaling. Hair grew back but the steroid dose could not be reduced to <2.5 mg/d because periodic increases were needed when symptoms flared.

In November 2011, the cat was brought back to the veterinary clinic because of worsening of the skin condition even though the owner had increased the prednisolone dose to 7.5 mg/d during the previous 3 weeks. The cat had also started shaking its head frequently and had a unilateral right nasal discharge. Major nasal discharge had not been a symptom previously reported by the owner. Cytologic analysis of the discharge showed large yeast bodies consistent with a *Cryptococcus* sp. interspersed among neutrophils, cocci, and rods.

The cat was treated with fluconazole, and prednisolone was slowly decreased to minimal doses to control the mural folliculitis. After consultation with the Alaska Office of the State Veterinarian and Division of Public Health, a nasal swab specimen was sent to the Centers for Disease Control and Prevention (CDC) (Atlanta, GA, USA) for confirmation and molecular typing. Since 2009, Alaska has participated in the CDC-led Pacific Northwest *C. gattii* working group, which has been interested in enhanced surveillance for *C. gattii* (*1**,**2*). CDC identified the isolate as *C. gattii* molecular type VGIII; this type is commonly reported in the southern United States, particularly California (*3*).

Given what is known about the potential for dispersal of *C. gattii* (*4*), the owner was extensively interviewed about travel history of the cat and household members, and any travelers to or visitors from California who may have brought items into the house. The cat was rescued as a stray at ≈1 year of age in California, and after traveling to Anchorage had lived as an indoor/outdoor cat without further travel. The family had not transported or received organic materials from California, except for an elongated (≈45 cm) seedpod. All plants and potting soil had been bought locally from national chain vendors. In May 2012, fifteen environmental samples, including soil from the yard, commercial potting soil, and a planter made from the seedpod brought from California, were taken from the home of the cat; all showed negative results for *C. gattii* when tested at CDC.

In humans, the average incubation period for *C. gattii* infection is 6 weeks–13 months (*5**–**7*). Therefore, case-patients are usually asked to recall potential exposures during the 13 months before symptom onset (*5*). Although most reported cases of *C. gattii* infection appear to be primary infections, infrequent reports of *C. gattii* infections in immunocompetent persons have described symptoms occurring several years after likely exposure, which suggests that *C. gattii* may have a greater capacity to remain dormant than believed (*8**–**10*). Incubation periods are not well described for animals but are generally reported as 2–11 months (R. Wohrle, pers. comm.). For either animal or human case-patients living in disease-endemic areas, precise incubation periods are likely incalculable because potential exposure to fungi is ongoing.

We suspect that *C. gattii* infection in this cat resulted from a distant exposure in California. Although the cat left California >8 years ago, it recently became immunosuppressed by medication that may have altered host factors, thus enabling latent fungi to clinically manifest. Results of limited environmental sampling in Alaska were negative. However, the molecular subtype supports California as a source, although the possibility that something or someone from California could have acted as a fomite could not be ruled out. Reactivation of a previous infection could also not be ruled out because the cat was a stray for the first year of its life.
